# Data pertaining to aberrant intracellular calcium handling during androgen deprivation therapy in prostate cancer

**DOI:** 10.1016/j.dib.2022.108143

**Published:** 2022-04-17

**Authors:** Debbie O'Reilly, Tim Downing, Sana Kouba, Marie Potier-Cartereau, Declan J. McKenna, Christophe Vandier, Paul Buchanan

**Affiliations:** a DCU Cancer Research Group, DCU, Ireland; b National Institute Cellular Biotechnology, DCU, Ireland; c School of Nursing, Psychotherapy and Community Health, DCU, Ireland; d School of Biotechnology, DCU, Ireland; e Genomic Medicine Research Group, School of Biomedical Sciences, Ulster University, N.Ireland; f Department of Animal Physiology, UMR INSERM U 1069, Nutrition, Growth and Cancer (N2C), University of Tours, France

**Keywords:** Prostate cancer, Androgen deprivation, Store operated calcium entry, Voltage gated calcium channels, CaV1.3, Calcium channel blocker, Store operated calcium channels, ORAI STIM, ADT, Androgen Deprivation Therapy, AR, Androgen Receptor, CACNA1D, L-type Calcium Channel Gene, CaV1.3, L-type Calcium Channel Protein, CCB, Calcium Channel Blocker, CRPC, Castrate Resistant Prostate Cancer, ERG, ETS-Related Gene, HPRT1, Hypoxanthine Phosphoribosyltransferase 1, NSE, Neuron Specific Enolase, PCa, Prostate Cancer, ORAI, ORAI Calcium Release-Activated Calcium Modulator 1, Tg, Thapsigargin, SERCA, Sarco(endo)plasmic Reticulum Calcium-ATPase, SOCE, Store Operated Calcium Entry, STIM1, Stromal Interaction Molecule 1

## Abstract

The data generated here in relates to the research article “CaV1.3 enhanced store operated calcium promotes resistance to androgen deprivation in prostate cancer”. A model of prostate cancer (PCa) progression to castration resistance was employed, with untreated androgen sensitive LNCaP cell line alongside two androgen deprived (bicalutamide) sublines, either 10 days (LNCaP-ADT) or 2 years (LNCaP-ABL) treatment, in addition to androgen insensitive PC3. With this PCa model, qPCR was used to examined fold change in markers linked to androgen resistance, androgen receptor (*AR*) and neuron specific enolase (*NSE*), observing an increase under androgen deprivation. In addition, the gene expression of a range of calcium channels was measured, with only the L-type Voltage gated calcium channel, *CACNA1D*, demonstrating an increase during androgen deprivation. With CACNA1D knockdown the channel was found not to influence the gene expression of calcium channels, *ORAI1* and *STIM1*. The calcium channel blocker (CCB), nifedipine, was employed to determine the impact of CaV1.3 on the observed store release and calcium entry measured via Fura-2AM ratiometric dye in our outlined PCa model. In both the presence and absence of androgen deprivation, nifedipine was found to have no impact on store release induced by thapsigargin (Tg) in 0mM Ca^2+^ nor store operated calcium entry (SOCE) following the addition of 2mM Ca^2+^. However, *CACNA1D* siRNA knockdown was able to reduce SOCE in PC3 cells. The effect of nifedipine on CaV1.3 in PCa biology was measured through cell proliferation assay, with no observed change in the presence of CCB. While *siCACNA1D* reduced PC3 cell proliferation. This data can be reused to inform new studies investigating altered calcium handling in androgen resistant prostate cancer. It provides insight into the mechanism of CaV1.3 and its functional properties in altered calcium in cancer, which can be of use to researchers investigating this channel in disease. Furthermore, it could be helpful in interpreting studies investigating CCB's as a therapeutic and in the development of future drugs targeting CaV1.3.

## Specifications Table

**Table:** 

Subject	Cancer Research and Cell Biology
Specific subject area	Electrophysiology of store operated calcium in androgen resistant prostate cancer and its associated impact on disease cell biology.
Type of data	Graph Figure
How the data were acquired	Cell culture Protein fractionation qPCR – Roche Nano Lightcycler Western blot – Image measurement ImageJ Fura-2am fluorescent Calcium measurement using a Wallac 1420 Victor2 Microplate Reader Colony formation assay Statistical Analysis: GraphPad Prism Data Collection – qPCR and Calcium measurement data was collected from computer. Western blot images were scanned and analysed with ImageJ. All other data was recorded manually on an excel file and processed accordingly.
Data format	Raw Analyzed
Description of data collection	Androgen sensitive prostate cancer cell line, LNCaP, was subject to (1) DMSO vehicle or ADT Bicalutamide (2) 10 days (LNCaP-ADT) or (3) 2 years (LNCaP-ABL). In addition to androgen insensitive, PC3. All with or without treatment of calcium channel blocker, nifedipine. Extracted RNA and protein
	was used to measure expression via qPCR and Western blot respectively. Store operated calcium was measured via FURA-2am. Cell proliferation was assessed with WST-1 assay.
Data source location	Dublin City University, National Institute of Cellular Biotechnology and the School of Nursing and Human Science, Dublin, Ireland.
Data accessibility	Repository name: Mendeley Data Data identification number: 10.17632/d9vn7ygf3z.4 Direct URL to data: https://data.mendeley.com/datasets/d9vn7ygf3z/5
Related research article	**For an article published in Cell Calcium.** D. O'Reilly, T. Downing, S. Kouba, M. Potier-Cartereau, D.J. McKenna, C. Vandier, P. Buchanan. CaV1.3 enhanced store operated calcium promotes resistance to androgen deprivation in prostate cancer. Cell Calcium. Volume 103, May 2022, 102554. https://doi.org/10.1016/j.ceca.2022.102554

## Value of the Data

• This data expands our understanding into the role of altered store operated calcium in androgen resistant prostate cancer, as well as improving our knowledge on calcium channel blocker (CCB) sensitivity of voltage gated calcium channels such as CaV1.3 in disease states such as cancer.
• The outlined data can be used to support further investigations into altered store operated calcium in androgen resistant prostate cancer. In addition, it will aid in the development of future experiments investigating the mechanisms of CaV1.3 in disease.
• In addition, it also provides further insight into previous or future studies using calcium channel blockers as a target for CaV1.3 in disease.

## 1 Data Description

This data provides supporting information to the outlined article “CaV1.3 enhanced store operated calcium promotes resistance to androgen deprivation in prostate cancer” [1]. Raw and analysed data in excel format relating to this article is provided at Mendeley data in the same order as outlined in this article [2]. This includes gene expression CT data for all genes outlined as well as housekeeping. CaV1.3 protein expression data values as quantified by ImageJ. 340/380 florescence measurements overtime for all calcium traces. In addition to absorbance values of WST-1 at 440nM relating to cell proliferation and colony counts for cell survival assays.

**Fig. 1:**
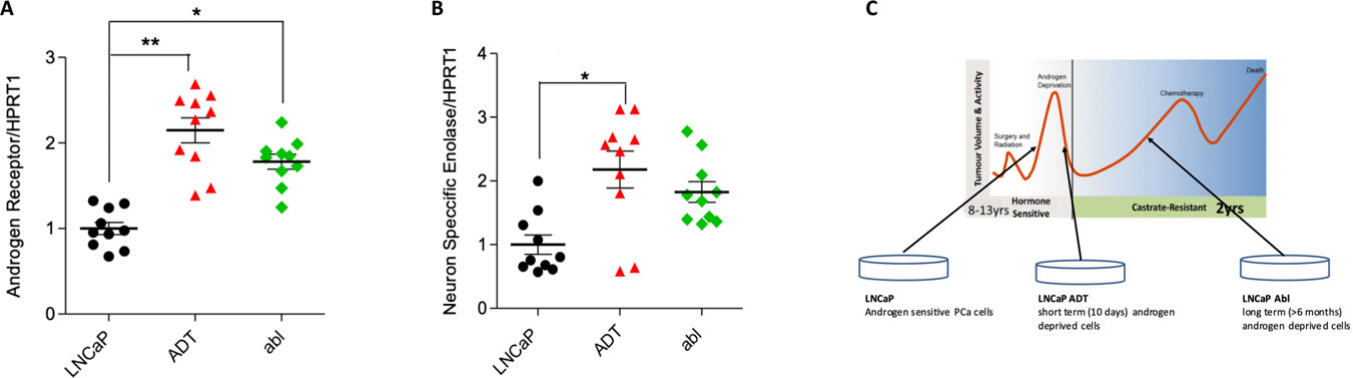
**Bicalutamide treatment increases expression of androgen resistance markers:** Genetic expression of **(A)** androgen receptor (N = 5, n = 10) and **(B)** neuron specific enolase (N = 5, n = 10), fold change against HPRT1 was assessed using qPCR in androgen sensitive LNCaP cells (black), LNCaP-ADT cells treated with 10µM bicalutamide (10 days) (red) and androgen insensitive long-term androgen deprived LNCaP-abl cells (green). **(C)** Summary figure displaying the associated stages of PCa disease that each model represents. Data normalised to LNCaP and analysed using Kruskal-Wallis with Dunn’s MCT. * P < 0.05, ** P < 0.01.

A model of disease progression during androgen deprivation therapy (ADT) was employed, representing untreated androgen sensitivity (LNCaP), early androgen resistance (LNCaP-ADT) and castrate resistant PCa (CRPC) (LNCaP-ABL). In addition to an androgen insensitive PCa cell line, PC3. With which the gene expression of androgen resistance markers were measured (Fig. 1) [3], [4], [5]. Androgen receptor (*AR*) expression was significantly increased during ADT treatment in LNCaP-ADT (p<0.01) and LNCaP-ABL (p<0.05) compared to untreated LNCaP (Fig. 1A). In addition, neuron specific enolase (*NSE*) was also significantly increased due to ADT in LNCaP-ADT (p<0.05), alongside a non-significant increase in LNCaP-Abl (Fig. 1B). The stages of PCa disease that the cellular model represents are summarised in Fig. 1C.

**Fig. 2:**
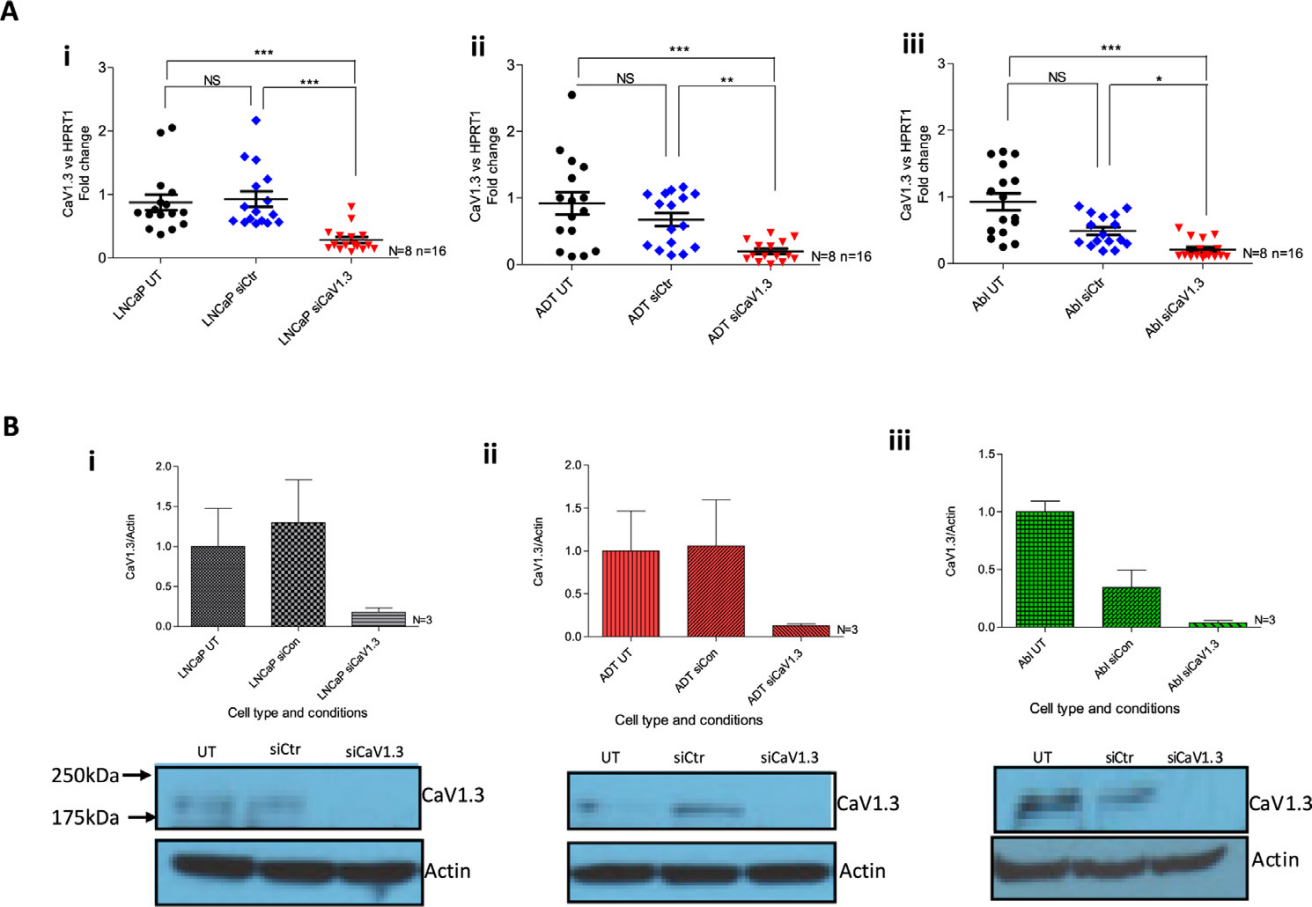
**Gene and protein expression of *CACNA1D* after knockdown. (A)** PCR analysis of CACNA1D expression fold change from HPRT1 in (i) LNCaP, (ii) LNCaP-ADT, (iii) LNCaP-Abl cells transfected with control siRNA (blue) or siRNA targeting CaV1.3 (red), normalised to untransfected cells (black) (Kruskil-Wallis, Dunn’s MCT, N = 8, n = 16). **(B)** Western blot analysis of expression of CaV1.3 fold change from Actin in (i) LNCaP, (ii) LNCaP-ADT, (iii) LNCaP-Abl cells transfected with control siRNA or siRNA targeting CACNA1D, normalised to untransfected cell of type (black) (Kruskil-Wallis, Dunn’s MCT, N = 3). * P < 0.05, ** P < 0.01, ***P < 0.001, NS not significant.

The outlined primary article investigated the effect of L-type calcium channel, *CACNA1D*/CaV1.3 (Gene or protein respectively) on SOCE using siRNA knockdown [1]. This was validated at a gene level for *CACNA1D* across the described PCa model (Fig. 2). Compared to transfected siRNA control, *siCACNA1D,* resulted in a significant reduction in *CACNA1D* gene expression in LNCaP (p<0.0001, Fig. 2Ai), LNCaP-ADT (p<0.001, Fig. 2Aii) and LNCaP-ABl (p<0.05, Fig. 2Aiii). The impact of *siCACNA1D* on CaV1.3 protein expression was determined with a reduction of >80%across all three cell lines (Fig. 2B).

**Fig. 3:**
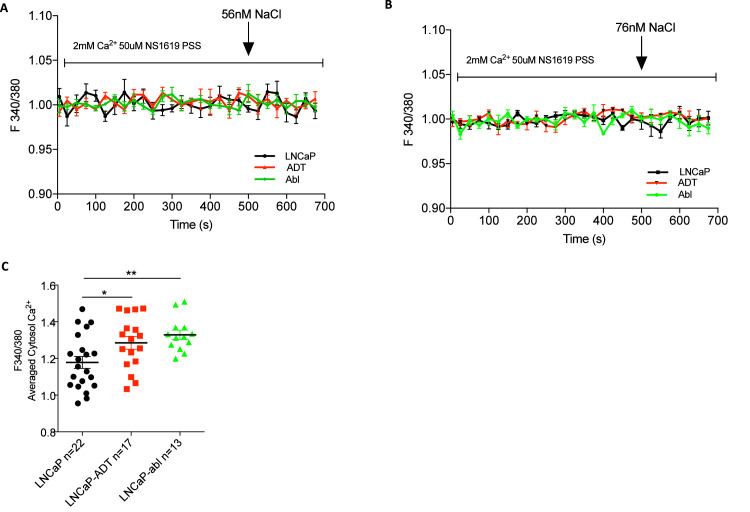
**NaCl osmolarity control for high potassium test and basal cytosolic calcium.** Fura-2am calcium traces over time of osmolarity controls where androgen sensitive LNCaP cells, LNCaP-ADT cells treated with 10µM bicalutamide (10 days) and androgen insensitive long-term androgen deprived LNCaP-Abl cells where depolarised with high external sodium concentrations of **(A)** 56mM NaCl or **(B)** 76mM NaCl (N = 3). **(C)** The average of the Basal cytosolic calcium traces was calculated in the same outlined cell models and displayed as a dot plot. N = 3.

The outlined primary article observed that high external potassium failed to mediate calcium influx following membrane depolarisation [1]. The addition of 56mM NaCl (Fig. 3A) and 75mM NaCl (Fig. 3B) were tested as osmolarity controls, finding no change in basal Ca^2+^ in any of the outlined PCa cell lines. Average basal cytosol calcium (Ca_c_^2+^) in 0mM PSS was also measured (Fig. 3C), observing that ADT treatment induced a significant Ca_c_^2+^ increase in both short term LNCaP-ADT cells (p<0.05) and long-term ADT, LNCaP-ABL cells (p<0.01).

**Fig. 4:**
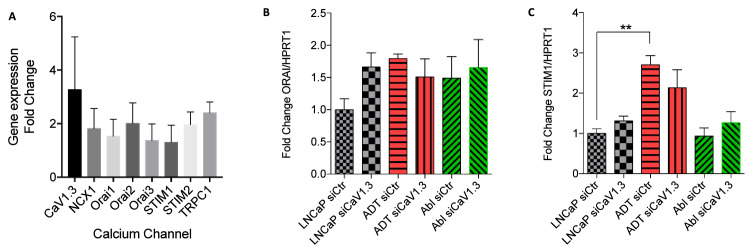
**Changes in store operated calcium channel expression under ADT and associated regulation by *CACNA1D* (A)** Gene expression of calcium channels implicated in store operated calcium after 10-day ADT assessed using qPCR against beta actin and displayed as fold change compared to untreated LNCaP (N = 3). **(B)***ORAI1* and **(C)***STIM1* gene expression assessed using qPCR against *HPRT1* in androgen sensitive LNCaP cells (black), LNCaP-ADT cells treated with 10µM bicalutamide (10 days) (red) and androgen insensitive long-term androgen deprived LNCaP-abl cells (green), transfected with either non-targeting siRNA (siCtr) or siRNA targeting *CACNA1D* (siCaV1.3) (N = 3, n = 6). Analysed using Kruskal-Wallis and Dunn’s multiple comparison post hoc test between cell types and treatments. * P < 0.05, ** P < 0.01, ***P < 0.001, NS not significant.

The gene expression of a range of store operated calcium channel families was measured in LNCaP-ADT compared to untreated LNCaP (Fig. 2A). The expression of all calcium channels was increased due to ADT, but *CACNA1D*, was found to be predominately expressed with a 2-fold increase on average compared to other calcium channels (Fig. 4A). *ORAI1* and *STIM1 gene expression was measured individually due to their role in store operated calcium (SOC)* under ADT in PCa [6]. No change was found in *ORAI1* under ADT, nor did *siCACNA1D* have any effect (Fig. 4B). *STIM1* was observed to have a significant increase in early-stage ADT treatment (LNCaP-ADT) versus untreated LNCaP (p<0.001, Fig. 4C), which was lost in androgen insensitive LNCaP-ABL. *siCACNA1D* failed to have any effect on STIM1 gene expression across all conditions.

**Fig. 5:**
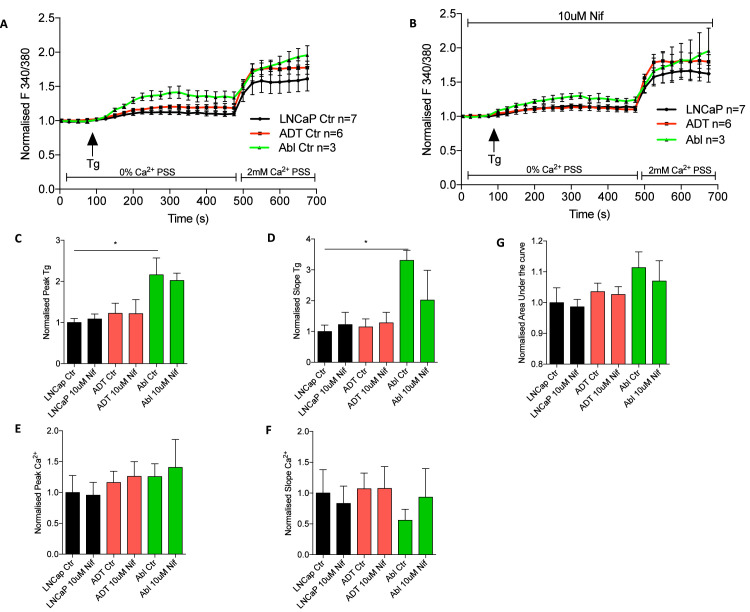
**Nifedipine has no significant effect on store operated calcium in prostate cancer cells either with or without ADT:** Trace of store operated calcium determined by Fura 2-AM ratiometric analysis over time(s) in untreated LNCaP or ADT treated LNCaP-ADT and LNCaP-Abl with either **(A)** DMSO control or **(B)** 10µM Nifedipine, analysed using two-way ANOVA Dunett’s MCT. Bar charts displaying the calculated **(C)** Tg peak, **(D)** Tg slope, **(E)** calcium peak, **(F)** calcium slope and **(G)** Area under the curve normalised to LNCaP Ctr. Analysed using Mann-Whitney test. * P < 0.05.

The linked publication demonstrated using *siCACNA1D* that increased CaV1.3 under ADT drove store operated calcium entry (SOCE) [1]. As a known CaV1.3 inhibitor the calcium channel blocker (CCB), nifedipine, was used to test its ability block CaV1.3 mediated SOCE (Fig. 5). Calcium traces displayed an increase in store release following thapsigargin (Tg) treatment across all three cell lines that was similar in the presence (p<0.0001) or absence (p<0.0001) of nifedipine (Fig. 5A+B). Further analysis found that ADT significantly increased the Tg peak (Fig. 5C) in long term CRPC (LNCaP-Abl, p<0.005), which was not altered by nifedipine treatment. This was also mimicked in the analysis of the Tg-induced slope, with an increase in LNCaP-Abl which is no altered by nifedipine (p<0.05, Fig. 5D). Following the addition of 2mM Ca^2+^ a significant increase in SOCE was witnessed again across all three cell lines in both the DMSO control (P<0.0001) and nifedipine treated (p<0.0001) (Fig. 5A+B). Comparing the Ca^2+^ peak a nonsignificant stepwise increase was observed in short (LNCaP-ADT) to long term ADT (LNCaP-Abl) compared to untreated LNCaP (Fig. 5E). No significant differences were observed in cells between DMSO control and nifedipine treatment. Analysis of Ca^2+^ entry slope found no significant difference between any cell type either with ADT or nifedipine (Fig. 5F). Calculation of the total change in Ca^2+^ through SOC was measured by area under the curve analysis, here a step wise increase was observed from short (LNCaP-ADT) to long term (LNCaP-Abl) ADT treatment, but this failed to reach significance (Fig. 5G). Furthermore, nifedipine was found to have no significant effect.

**Fig. 6:**
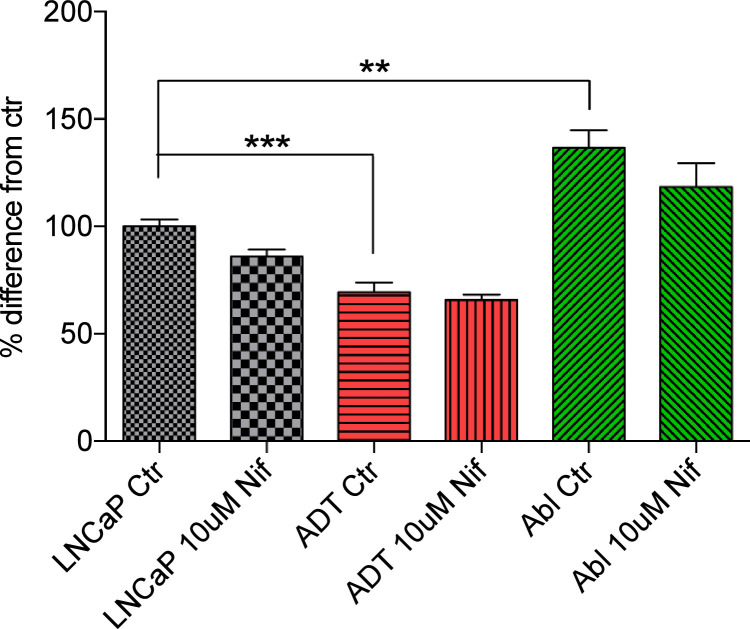
**Cell proliferation in prostate cancer cells is not inhibited by nifedipine:** Cell proliferation was assessed following treatment with 10µM nifedipine or DMSO control (N = 5, n = 15) in androgen sensitive LNCaP cells (black), LNCaP-ADT cells treated with 10µM bicalutamide (10 days) (Red) and androgen insensitive long-term androgen deprived LNCaP-abl cells (Green). Fold change was calculated compared to untreated LNCaP and displayed as a bar chart. Analysed using Kruskil-Wallis significance test and Dunn’s multiple comparison post hoc test between cell types and treatments. * P < 0.05, ** P < 0.01, NS not significant.

The effect of nifedipine compared to DMSO control on cell proliferation was tested (Fig. 6). The outlined cell line model displayed a significant reduction in LNCaP-ADT proliferation verses untreated LNCaP in the DMSO control (p<0.001), with nifedipine displaying no effect. Long term ADT CRPC cell line LNCaP-Abl displayed a significant increase in cell proliferation (p<0.01) but nifedipine did not have any impact.

**Fig. 7:**
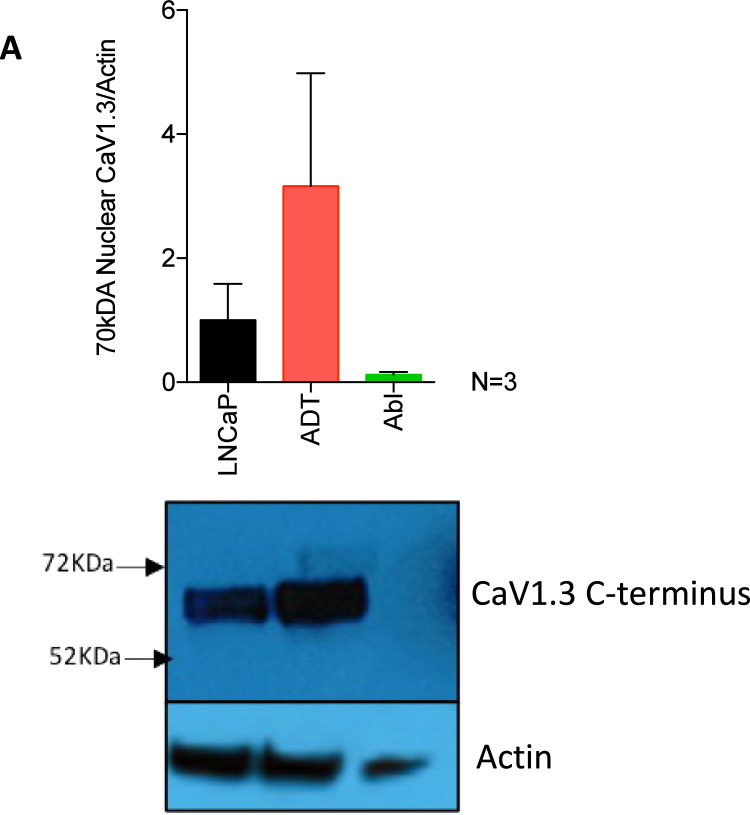
**Loss of CaV1.3 C-terminus expression in castrate resistant prostate cancer. (A)** Bar graph showing the quantification of nuclear CaV1.3 C-terminus protein expression in the untreated LNCaP or short (LNCaP-ADT) and long (LNCaP-ABL) term ADT and **(B)** associated blots (N = 3).

CaV channels are known to produce a C-terminus that can influence gene transcription [7], using protein fractionation the presence of the CaV1.3 C-terminus in the nucleus was measured (Fig. 7). Its presence was found in both LNCaP and early-stage ADT resistant LNCaP-ADT, the latter of which displayed a 3-fold increase. In castrate resistant LNCaP-ABL, expression was completely lost. Note, this is preliminary data without a nucleus control so further work is require to confirm this.

**Fig. 8:**
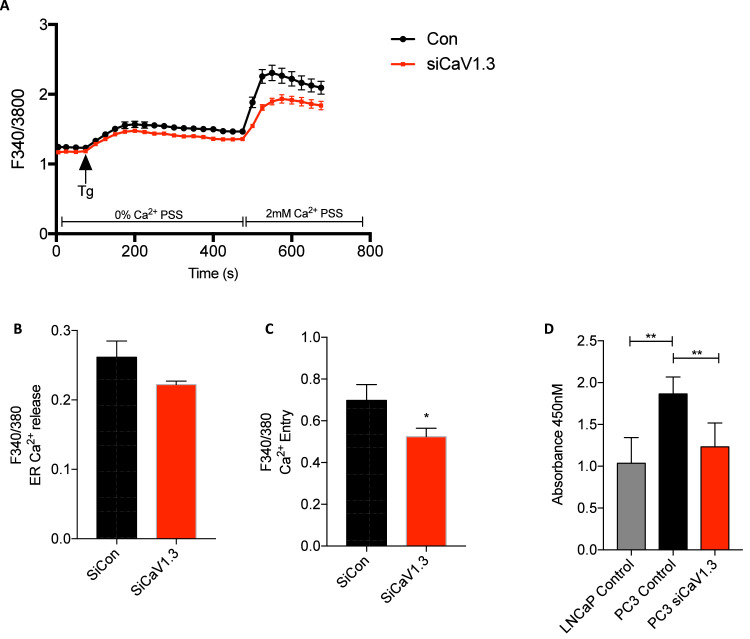
***siCACNA1D* reduces calcium entry in PCa cell line PC3. (A)** Store operated calcium trace determined by Fura 2-AM ratio metric F340/F380 analysis of calcium concentration over time(s) in PC3 cells treated with either sicontrol or siCaV1.3. Bar graphs representing average change in **(B)** Tg peak or **(C)** calcium peak compared to associated baseline reading. Analysed using Wilcoxon test between siCon and *siCANCA1D*. **(D)** Cell proliferation of LNCaP or PC3 measured by WST-1 absorbance at 450nM, with PC3 cells treated with siControl or siCACNA1DAnalysed by Mann-Whitney T-test. All N = 3.

Androgen insensitive PC3 cell line was used to measure the role of CaV1.3 on SOC and proliferation using *siCACNA1D*. PC3 cell lines displayed a significant increase in store release under Tg treatment (P<0.0001) followed by a significant increase in SOCE (p<0.0001) (Fig. 8A). Following *CACNA1D* siRNA knockdown store release was decreased but did not reach significance (Fig. 8B), however SOCE did (p<0.05, Fig. 8C). PC3 proliferation was significantly increased compared to androgen sensitive LNCaP (p<0.01, Fig. 8D), which was reduced by *siCACNA1D* (p<0.01, Fig. 8D) to levels observed with LNCaP.

Bioinformatic analysis was conducted in the original paper using the samples from the *Taylor et al* data set [8]. These samples had been spilt into adjacent, primary, and metastatic for which the numbers are outlined (Table 1). The numbers used in each cohort for Gleason score, hormone therapy and ETS-related gene (*ERG)* status are all outlined in the table.

## 2 Experimental Design, Materials and Methods

### 2.1 Tissue culture

The human androgen receptor (AR) positive PCa cell line LNCaP (ATCC # CRL-1740) was used as an androgen sensitive untreated control. Two androgen resistant sublines were developed using anti-androgen, 10µM bicalutamide (Sigma). LNCaP-ADT was treated with ADT for 10 days, the timepoint associated with significant AR and neuroendocrine gene expression linked to treatment resistance (Fig. 1) [3], [4], [5]. Alongside a previously published 2 year ADT insensitive CRPC, LNCaP-ABL [9]. In addition to androgen independent cell line PC3. All lines were maintained in RPMI (Gibco) with 10% fetal bovine serum (Gibco) in a humidified incubator at 37 °C and 5% CO_2_. In addition, they were mycoplasma negative and had their origin confirmed via short tandem array (STR) profiling.

### 2.2 Calcium channel blocker Nifedipine

Nifedipine stock concentration of 100 mM was made up by adding 34.63 mg Nifedipine (Acros Organics) to 1 ml DMSO. Media containing nifedipine was produced by adding 0.1 µl/ml of 100mM stock Nifedipine to cell culture media above resulting in a final concentration of 10 µM Nifedipine.

### 2.3 siRNA transfection

siRNA was received as lyophilised powder and reconstituted with PCR grade H_2_O to a concentration of 5µM, aliquoted into 20 µl and stored at -20 °C. Cells were seeded into the relevant plates and allowed to grow to 80% confluency in relevant media, without antibiotic. The media was then removed and replaced with Opti-MEM™ (Gibco), reduced serum media. The transfection mix was made up using the relevant volumes for the plate outlined in manufactures manual of the Dharmacon transfection reagent 3 (Cat no: T-2003-02) along with Dharmacon ON-TARGETplus siRNA Non-targeting (Negative control, Cat No: D-001810-10-05) and *CACNA1D* siRNA (776, Cat No: L-006124-00-0005.The transfection mix was added to each well as required. The cells were incubated at 37 °C with 5% CO_2_ for 48 hours prior to RNA extraction. Knockdown efficiency was assessed by qPCR and western blotting with a minimum of 70% reduced genetic expression and 87% protein expression observed.

**Table 1:** Sample details used for bioinformatic analysis. PCa primary and metastatic tumour and match normal samples were acquired from radical prostatectomy. **(A)** Tumour type vs origin. This sample set contained expression information from a total of N=176 samples of which n=130 where primary cancers and n=18 metastatic (The 19 metastatic samples were from lung, neck, spine, bladder, testes, bone, brain and colon tissue). A subset of primary cancers also had n=28 paired adjacent normal samples. **(B)** Sample size and tumour type for each hormone therapy group used. **(C)** Sample source and Combined Gleason score showed that most samples had scores of 6 or 7 than 8 or 9. **(D)** Sample source and ERG status showed that most were ERG-negative (73%) and a minority were ERG-positive (27%) and that these proportions were similar in both metastatic (78% and 22%) and primary tumours (72% and 28%). **(E)** Cohort ADT hormone therapy treatment and ERG status numbers.

A				
Origin	ADJACENT	MET	PRIMARY	
Asian	1	0	2	
Black Hispanic	0	0	3	
Black Non-Hispanic	0	1	24	
Unknown	2	0	4	
White Hispanic	0	2	0	
White Non-Hispanic	25	15	97	

B				

	Tumour Type			
Hormone therapy	Adjacent	Metastatic	Primary	Total

Post-ADT	0	7	3	10
ADT	2	8	16	24
No Treatment	26	3	111	114

C				

Gleason Score	ADJACENT	MET	PRIMARY	

6	12	0	41	
7	13	2	73	
8	1	3	8	
9	1	4	7	
No data	1	9	1	

D				

ERG Status	Adjacent	MET	Primary	

Negative	19	14	78	
Positive	3	4	30	
Flat	6	0	22	

E				

Hormone therapy	ERG status	Totals		

Post-ADT/ADT	Negative	23		
	Positive	9		
	Flat	2		
	Subtotal	34		

No treatment	Negative	70		
	Positive	26		
	Flat	18		
	Subtotal	114		

Total		148

**Table 2:** PCR primers. The primers used for the generation of the outlined data are listed with gene name, full name and forward and reverse primers.

Table 2 – PCR primer sequences		
Gene	Gene Name	Sequence
AR	Androgen Receptor	F- TTTTTCTAAGACCTTTGAACT R- TCTGTGGAAGTCGCCAAGTT
CaV1.3	Voltage-dependent L-type calcium channel subunit alpha-1D	F- ATGTAGGAGTGGCTGGGTTG R- CCATGGTGATGCACTGAAAC
ENO1	Neuron Specific Enolase 1	F- CTGTGGTGGAGCAAGAGAAA R- ACACCCAGGATGGCATTG
NCX1	Sodium/calcium exchanger 1	F- CGTCGCACTTGGAACATCAG R- CCATTGGCTGCGTGGTAGAT
ORAI1	Calcium release-activated calcium channel protein 1	F- AGGTGATGAGCCTCAACGAG R- CTGATCATGAGCGCAAACAG
ORAI2	Calcium release-activated calcium channel protein 2	F- TGGATTACCGGGACTGGGT R- CTCGCTGATGGAGTTCAGGT
ORAI3	Calcium release-activated calcium channel protein 3	F- CCACGTACCGGGAGTTCG R- ACTCGTGGTCACTCTCCAGC
STIM1	stromal interaction molecule 1	F- GCCCTCAACATAGACCCCAG R- TCCATGTCATCCACGTCGTCA
STIM2	stromal interaction molecule 2	F- TTGGACCCTTGAAGACACTCT R- CCAGTTATGAGGTGGGCGTG
TRPC1	transient receptor potential channel 1	F- TTACTTGCACAAGCCCGGAA R- CTGCTGGCAGTTAGACTGGG

### 2.4 RNA extraction and cDNA synthesis

Total ribonucleic acid (RNA) was extracted using High Pure RNA Isolation Kit (Roche) as per the supplied instructions. Total RNA was quantified using NanoDrop 1000™ (Thermo Scientific). 1µl RNA extraction was used to give quantity in ng/µl. Purity of RNA was assessed using absorbance 260nm and 280nm (A260/A280) with a ratio of 1.8-2.2 accepted as pure RNA. Complimentary deoxyribonucleic acid (cDNA) was synthesised from RNA extractions using Transcriptor first strand cDNA synthesis kit (Roche) using 2.5µM Anchored-oligo primer and 60µM random hexamer primer. Producing 1ug cDNA in 20µl which was stored at -20 °C.

### 2.5 Primer design

All primers were designed using primer 3 (https://primer3.ut.ee/), using Exon spanning sequences where possible. Oligo Calc (http://biotools.nubic.northwestern.edu/OligoCalc.html) was used to detect any potential hairpin formations or self-annealing sites. All primers were acquired from Sigma Aldrich as lyophilised powder which were reconstituted with PCR grade H_2_O to stock concentration 100µM, from which working stock primers (5µM) were made.

### 2.6 Real time polymerase chain reaction (qPCR)

Master mix was prepared for multiple reactions by adding 5µl FastStart Essential DNA Green Master (Roche), 1µl forward primer (0.5µM) and 1 µl reverse primer (0.5µM) per reaction. 2µl cDNA (50ng) was added to each reaction alongside the 7µl of the master mix and the volume made up to 10µl with PCR grade H2O. The PCR strip was placed in the LightCycler Nano™ (Roche) which was programmed to run as outlined in the FastStart Essential DNA Green Master protocol, with the annealing temperature adjusted according to the primer melting temperature. The housekeeping gene, Hypoxanthine Phosphoribosyltransferase 1 (*HPRT1),* was used to determine the relative expression of each gene. The relative gene expression data was analysed using the 2-ΔΔCT method. The sequences the PCR primers used through are included (Table 2).

### 2.7 Cellular fractionation and protein quantification

To determine the cellular location of expressed proteins a cell fractionation kit was used (Abcam). The kit extracted three fractions, the cytosolic fraction, the membrane fraction, and the nuclear fraction. Cells were grown on 100mm cell culture dishes to approximately 6*10^6^ cells and processed as stated in the provided manual. Protein was quantified using the Pierce BCA Protein Assay Kit (Thermo Scientific). Protein samples were diluted 1:10 in PBS and 25µl were plated in triplicate. Working reagent (WR) was made up by mixing 50 parts of BCA Reagent A with 1 part BCA Reagent B. 200µl of WR was added to each well and the plate was incubated for 30mins at 37 °C. The absorbance was detected at 562nm. The standards were used to create a standard curve from which the concentration of each protein sample was calculated

### 2.8 Western blot

Protein lysates were separated on a Bolt Bis-Tris Plus pre-cast Gels (4-12%, Invitrogen) using the Mini Gel Tank (Invitrogen). In a microcentrifuge tube 50µg total protein was added to 5µl Bolt LDS sample buffer (Invitrogen) and 2µl Bolt reducing agent (Invitrogen). The total volume was made up to 20µl with sterile dH2O. The protein sample was heated at 70 °C for 10mins to denature. The samples were loaded into the relevant wells and the tank filled with x1 Bolt MOPS SDS running buffer (Invitrogen). The tank was connected to the electrical supply and run at 200V for 30mins. The gel was then transferred to a PVDF membrane (methanol activated) 0.4µm in Transfer buffer (Bolt Transfer buffer (Invitrogen) with Bolt Antioxidant (Invitrogen) at 20V for 60mins. The membrane was incubated in blocking buffer (Tris buffered saline, 0.1% tween 20 and 5% milk) for 1 hour at room temperature, followed by three 5 minutes washes in TBST. The membrane was subsequently incubated with CaV1.3 C-terminus antibody (Abcam, 1:500 dilution, mouse monoclonal, AB-84811) in TBST containing 5% milk over night at 4 °C with gentle agitation. Followed with a TBST wash before 1hr incubation with secondary HRP-Anti Mouse (HRP-Anti-Mouse, BD Pharmigen, Goat Polycloncal,1:1000). The membrane was then incubated in 1:4 dilution of Supersignal West Dura Chemiluminescent Substrate (Thermo Scientific) with gentle agitation for 1min at room temperature. Afterwards it was exposed to a piece of X-ray film in a cassette in the dark room before placed in developer solution, followed by fixer. Resultant blots where scanned and analysed using ImageJ.

### 2.9 Calcium measurements

Cells were grown in 6 well dishes with relevant test conditions for experimental parameters. Cells were then removed, counted, and resuspended in a tube in OptiMEM™ low serum media at a concentration of 5*10^5^ cells/ml. Fura 2-AM was added to the tube to a final concentration of 2uM and incubated at RT in the dark for 45mins. The cells were centrifuged at 8000g for 5mins and the supernatant was discarded. The pellet was then resuspended in 1ml OptiMEM™ and incubated in the dark for 30mins. This process was repeated, and the cells were incubated in the dark for 45mins to allow complete de-esterification of the dye. Tubes were then centrifuged and the pellet was resuspended in 500µl physiological saline solution PSS 0% Ca^2+^ (NaCl 142mM, MgCl_2_ 1mM, KCl 4mM, D-glucose 11.1mM,1 mM EGTA and HEPES 10mM pH balanced to 7.4 with NaOH). From this100µl was added to each well of a black walled 96 well plate to give 1*10^5^ cells/well. Measurements were made using the VICTOR multilabel plate reader with excitation wavelengths set at 340 and 380nm and the emission wavelength set at 510nm with measurements recorded every 5s. The plate reader was preloaded with the Tg and Ca^2+^ solutions which were injected at a volume of 20µl/well. Baseline Ca^2+^ levels were determined for 100s then Tg was injected at final concentration of 4µM and recorded for 400s after which CaCl_2_ was injected at a final concentration of 2mM with measurements recorded for a further 200s. Tg results in the depletion of the ER Ca^2+^ stores by inhibiting the action of SERCA. Introducing Ca^2+^ to the extracellular fluid allows us to see the level of extracellular Ca^2+^ that is taken into the cell as a result of ER store depletion. The addition of high external potassium of 56 mM KCl (K60) or 76 mM KCl (K80) was used to induce membrane depolarization. Associated osmolarity controls of 56 mM NaCl (Na196) or 76 mM NaCl (Na216) was used. These measurements where contacted in PSS (in mM): NaCl 140, MgCl2 1, KCl 4, CaCl2 2, D-glucose 11.1 and HEPES 10, adjusted to pH 7.4 with NaOH, as well as potassium channel blocker NS1619 (50µM) to induce membrane hyperpolarised.

### 2.10 Cell proliferation

Proliferation was assayed using a Wst-1 assay kit (Roche), measuring the formation of formazan through the enzymatic cleavage of tetrazolium salt by the mitochondrial dehydrogenases present in viable cells. Cells were seeded into 96 well plates 100µl per well at a density of 5*10^5^ cells per ml and placed into the incubator at 37 °C with 5 % CO_2_ for 48 hours, afterwards cells were treated with test conditions and controls as required. At the treatment end point 10µl of Wst-1 reagent was added to each well and replaced back into the incubator for 4 hours. The plate is then placed into VICTOR multilabel plate reader and gently shook for 1min. before absorbance was recorded at 440nm. The background control was also recorded at 440nm containing Wst-1 in media. The averaged blank measurement was subtracted from each measurement and the triplicate average was used for analysis. The experiments were compared to the LNCaP parental control cells, with the results presented as percentage change.

### 2.11 Statistical analysis

All graphs were prepared using Prism (Graphpad software, USA). Results are expressed as mean+/-s.e.m unless otherwise stated. Non-parametric tests were used for non-normal sample distributions and N<10. Non-parametric tests between two groups used a Mann-Whitney test or for multiple groups a Kruskal-Wallis with Dunn's MCT. The statistical tests employed are described in the figure legend, with annotations as *P<0.05, **p<0.01, ***p<0.001. All results are generated from at least three independent experiments denoted by the N number, with the total number of individual repeats within each denoted by the n number.

## Ethics Statements

No ethics required to produce this data.

## CRediT Author Statement

**Debbie O'Reilly:** Methodology, Formal analysis, Writing – original draft, Writing – review & editing, Visualization; **Tim Downing:** Writing - original draft, Writing – review & editing; **Sana Kouba:** Methodology, Resources, Formal analysis, Visualization; **Marie Potier-Cartereau:** Writing – review & editing; **Declan J. McKenna:** Methodology, Resources, Writing – original draft, Writing – review & editing; **Christophe Vandier:** Methodology, Resources, Writing – review & editing; **Paul Buchanan:** Conceptualization, Methodology, Formal analysis, Resources, Data Curation, Writing – original draft, Writing – review & editing, Visualization, Supervision, Project administration, Funding acquisition.

## Declaration of Competing Interest

The authors declare that they have no known competing financial interests or personal relationships that could have appeared to influence the work reported in this paper.

## Data Availability

Data pertaining to aberrant intracellular calcium handling during androgen deprivation therapy in prostate cancer (Original data) (Mendeley Data).
